# Defining the Nature of Augmented Feedback for Learning Intraosseous Access Skills in Simulation-Based Health Professions Education

**DOI:** 10.7759/cureus.41869

**Published:** 2023-07-14

**Authors:** Julia Micallef, Dale Button, Alvaro Uribe Quevedo, Christopher McClatchey, Lindsey King, Adam Dubrowski

**Affiliations:** 1 Health Sciences, Ontario Tech University, Oshawa, CAN; 2 Paramedicine, Durham College, Oshawa, CAN; 3 Software and Informatics, Ontario Tech University, Oshawa, CAN; 4 Paramedicine, Region of Durham Paramedic Services, Whitby, CAN

**Keywords:** delphi method, design-thinking, augmented feedback, design-based research, simulation in healthcare

## Abstract

In the field of health professions education, acquiring technical skills involves three stages: 1) receiving instructions, 2) engaging in practice, and 3) receiving feedback. Simulation serves as a valuable tool that encompasses all three stages, enhancing the effectiveness of health professions education. This work focuses on feedback, which can be categorized as intrinsic (perceived by the learner through their senses) or augmented (provided by an external perspective). Augmented feedback can take the form of knowledge of results (information regarding the outcome) or knowledge of performance (information about the actions leading to the outcome). The overall objective of this work was to evaluate the perceived efficacy of these types of feedback in learning technical skills using a simulation, specifically an intraosseous access simulator, among advanced care paramedics. The primary focus of this article and the initial step towards achieving the aforementioned objective of this work was to determine the possible knowledge of results and knowledge of performance that paramedic facilitators could offer to advanced care paramedics during the use of an existing intraosseous access simulator. This research was conducted following the design-based research framework, employing a combination of design thinking and Delphi methods to generate a comprehensive list of augmented feedback, in both the form of knowledge of results and knowledge of performance, that can be provided to advanced care paramedics while learning intraosseous access skills through a simulator. The design thinking session was carried out to generate an initial inventory of augmented feedback, which was then refined through two rounds of Delphi consensus-building with paramedic experts. This process resulted in an eight-step list of feedback for knowledge of results and knowledge of performance that can be delivered to advanced care paramedics by paramedic facilitators using an intraosseous access simulator.

## Introduction

Psychomotor skills are movement tasks that contain both cognitive and motor processes. These processes, in turn, often lead individuals to learn and manipulate the environment around them [1]. In the context of health professions education (HPE), psychomotor skills are often referred to as technical skills, being tasks that are performed by health professionals for the patient [2]. In HPE, learning technical skills involves three stages that ensure that both the cognitive and motor elements are understood: 1) instructions, 2) practice, and 3) feedback [2-4]. Instructions comprise the information provided to the learners about the skills and mechanical principles that are needed to perform the task [1]. Practice is an active process in which an individual attempts to perform a task with the intent of acquiring a new skill so that there is a permanent change in habit [1]. Finally, feedback can be defined as information that is given before, during, and after an action has occurred [1]. Feedback is typically divided into two categories: 1) intrinsic feedback and 2) augmented feedback. Intrinsic feedback is defined as the information that is naturally understood and gathered through our senses [1]. For example, a basketball player can see if they made their shot or feel if their throw was off. Augmented feedback supplements intrinsic feedback by providing information about the movement sequence or outcome from an external point of view [1]. Augmented feedback can be further subdivided into two main dimensions: knowledge of results (KR) and knowledge of performance (KP). KR is the feedback that is given regarding the outcome of the overall goal of the action. Using the same basketball example, the player’s coach may provide feedback on the parabola of the ball after it was shot or where the ball hit the backboard to provide an explanation as to why the ball did or did not go into the net. KP is the feedback provided regarding the action movement that leads to the outcome [1]. This is where the basketball coach would provide feedback regarding the form of the player while they were making the shot.

Simulation-based health professions education (SBHPE) uses simulators to allow individuals in health care professions to practice clinical procedures in a safe and controlled environment [5]. In this context, simulation can employ models, actors, animal parts, digital technologies, supplementary materials, and scripts to create an immersive, replicable, and standardized learning environment [5]. Therefore, using SBHPE to amplify HPE can be an effective tool that encompasses all three stages of learning technical skills.

Concerning feedback, it is unknown which type is most effective, especially within the context of SBHPE with advanced learners [1]. Therefore, the overarching aim of this work was to assess the perceived effectiveness of KP, KR, and intrinsic feedback in learning technical skills with advanced care paramedics (ACPs). We have selected intraosseous (IO) vascular access because it is a commonly utilized skill by ACPs, it has clear procedural steps, and we had access to a previously developed IO access simulator [6]. Before the perceived effectiveness could be investigated, we first had to determine what KP and KR could be provided specifically for learning IO access using the developed IO access simulator. Therefore, the objective of this article is to share the methods utilized and the results of determining what KP and KR can be provided in the context of learning IO access by ACPs.

## Materials and methods

The development of an advanced IO access simulator using 3D printing and silicone work was described in a previous report [6]. It is this IO access simulator that will be used, alongside feedback from a quality and development facilitator with an ACP certification, to assess the perceived effectiveness of KP, KR, and intrinsic feedback by ACPs from the Region of Durham Paramedic Services (Whitby, Ontario). However, before the perceived effectiveness can be investigated, we had to determine what KP and KR can be provided in the context of learning IO access using the advanced IO access simulator. The ethics for this project was exempted by the Ontario Tech University Research Ethics Board as per Tri-Council Policy Statement (TCPS2) Article 6.1 Issue Number 16892 as a protocol development study. The study as conducted at the Region of Durham Paramedic Services building (Whitby, Ontario).

**Figure 1:**
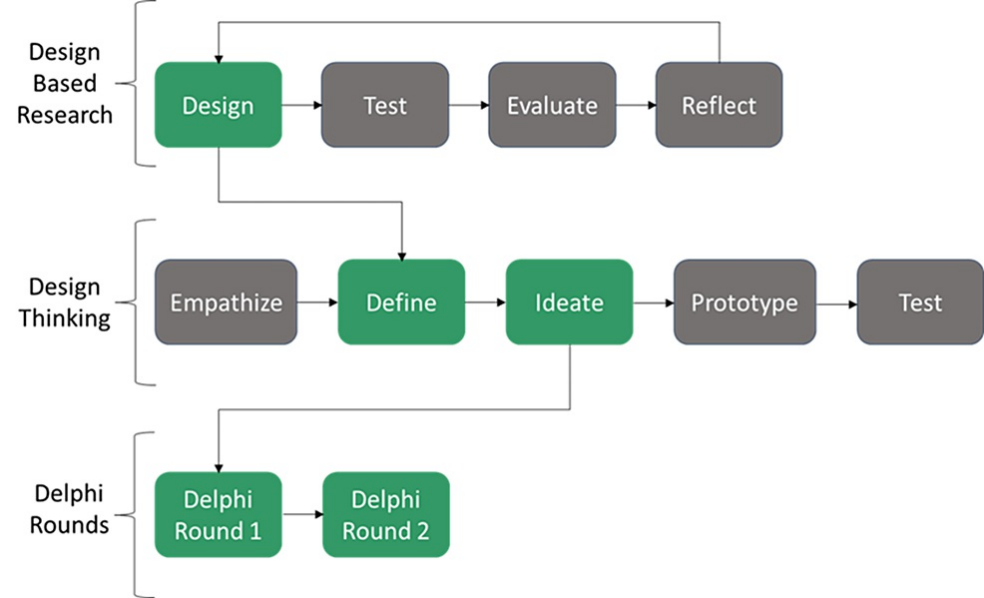
Situating the design thinking and Delphi methods within the design-based research framework

This work will be accomplished following the design-based research (DBR) framework, specifically within phase 1, which is the design phase. This phase will be accomplished using a hybrid of design thinking and Delphi methodology. Shown in Figure 1 below is how the methods will be situated within the DBR framework, with the phases in the green boxes being the phases utilized in this work.

Design-based research 

In order to determine what KP and KR can be provided to ACPs when learning IO access using our previously developed IO access simulator, we followed the DBR framework. DBR is an educational framework that explains an iterative process focusing on the collaboration of researchers, stakeholders, and end-point users to generate solutions (i.e., resources) that can be applied to specific learning contexts [7,8]. DBR contains four iterative phases: 1) design, 2) test, 3) evaluate, and 4) reflect [9]. The design phase involves developing a solution that addresses both the theoretical and practical concerns of the problem [8,10]. The test phase involves implementing the solution in a real-world setting [11]. The evaluate phase evaluates the effectiveness of the solution using evidence from endpoint users’ learning [12,13]. Finally, the reflect phase involves a retrospective analysis of the DBR methodology and methods used in the prior phases [12,14]. To achieve the desired outcomes, this work focuses on the design phase (Figure 1). The methods used to accomplish this phase are a hybrid of design-thinking (DT) and Delphi.

DT and Delphi hybrid

The combination of DT and Delphi used to achieve the objective of this work is pictured in Figure 1. During the DT session, the define stage was conducted to determine what the objectives of learning IO access are, and the ideate stage is where we outline what augmented feedback, in the form of KP and KR, can be provided to learners when using the IO access simulator. The feedback determined from the ideate stage of the DT session was then put through Delphi rounds in order to gain consensus on what KP and KR can be provided to ACPs when learning IO access using the IO access simulator.

DT process 

DT is a flexible and collaborative problem-solving process consisting of five stages: 1) empathize, 2) define, 3) ideate, 4) prototype, and 5) test [15]. The main goal of the empathize stage is for the researchers to get an understanding of who the stakeholders and end-point users are [15]. The define stage aims to pinpoint the needs and problems of the stakeholders and end-point users [15]. The ideate phase involves brainstorming possible solutions to the problem, which are then created in the prototyping phase [15]. The final test phase involves testing the proposed solutions to see if they meet the needs of endpoint users and stakeholders [15]. To fit into the scope of this work, we will just be focusing on the defining and ideating stages of DT. The DT session was held in person at the Region of Durham Paramedic Services building (Whitby, Ontario) with two members of the research team (Julia Micallef and Adam Dubrowski), a total of four participants (n=4); one ACP instructor (n=1), one ACP student (n=1), and two working ACPs (n=2). These participants were recruited via emails from the research team asking for participation. The inclusion criteria were that the participants had to be either a working ACP or an ACP student and had to be familiar with the IO access procedure. The one-hour session was conducted in the format of a focus group interview, facilitated by one of the researchers (JM), and guided using a PowerPoint presentation. The outputs from these phases then feed into the Delphi process.

Delphi process

The Delphi process is a structured group communication process that seeks to gather information and achieve consensus from a panel of experts using iterative survey questionnaires [16]. In general, the Delphi method process starts with the selection of a group of experts based on the topic being examined, referred to as the Delphi panel. Once all panelists are identified and confirmed, each is sent a survey with instructions to comment on each topic based on their personal opinion, experience, or previous research. They are then asked to return the surveys to the researcher, who groups the comments and prepares copies of the information. An agreement between the panelists is determined a priori, and the researcher determines if the agreement has been reached or if the content needs to be modified and resent for further deliberation by the panel. This is known as a Delphi round, and these rounds are repeated as many times as necessary to achieve a general sense of consensus [16]. For this work, we used an electronic survey (Google Forms), which was emailed to nine participants (n=9) consisting of ACPs from the Region of Durham Paramedic Services (n=6), paramedic educators (n=2), and a medical doctor (n=1). These participants were recruited via emails from the research team asking for participation. The inclusion criteria were that the participants had to be familiar with the IO access procedure. The survey was formatted so that each participant had to rate, on a 5-point Likert scale, the level of importance of the steps in the form of KP and KR indicated in the DT session. In addition to participants ranking on a 5-point Likert scale, there were also sections in the survey where they could provide comments. The cutoff criteria for the Delphi methods were set a priori, where a median above 3 and a standard deviation below 1 would be considered a consensus among the participants to keep the particular item on the list. Items with a median lower than 3 and a standard deviation lower than 1 would be rejected, and items with a median lower than 3 but a standard deviation higher than 1 would be revised based on comments and included in subsequent rounds, with one week to complete each round. There were no limits to the number of rounds, and the end of the consensus-building exercise was reached when all items scored a median over 3 and showed a standard deviation lower than 1. For this work, only two rounds were needed.

## Results

DT session 

The definition stage of the DT session was guided by asking the participants what the objectives for learning IO access are. This resulted in five main objectives: 1) to identify indications; 2) to use the appropriate tools; 3) to landmark correctly; 4) to ensure the IO needle is secured; and 5) to confirm the success of the IO access.

Next, during the ideation stage, the participants decided that the best course of action would be to use a previously developed skills checklist by one of the team members (DB), shown in Table 1, on how to perform an IO access and reword the steps so that they can be forms of augmented feedback (KP and KR). We condensed the 28 steps outlined in the skills checklist into seven steps that had a version of KP and KR for each step to ensure the feedback in each form was given in equal quantities. The results of the DT are shown in Table 2 below.

**Table 1:** Intraosseous access skills checklist

Completion requirements	Met or not met?
Ensures that adequate basic life support is performed	met/not met
Appropriate consent	met/not met
Appropriate infection control precautions	met/not met
Assembles and prepares necessary equipment	met/not met
Selects appropriate solution	met/not met
Checks solution for expiry, clarity, particulate, leaks	met/not met
Selects and flushes appropriate solution admin set	met/not met
Selects a site based on patient presentation/clinical need	met/not met
Places the patient on a resilient surface	met/not met
Leg externally rotated to display the medial aspect	met/not met
Landmarks 1-2 cm distal to tibial tuberosity on a flat portion of bone	met/not met
Cleans intended site with alcohol/betadine	met/not met
Swabs in a circular motion out from the injection site	met/not met
Inserts needle at approximately 90 degrees angled slightly away from the joint	met/not met
Uses firm twisting motion until “pop” is felt	met/not met
Unscrew the cap and removes the stylet directly into the sharps container	met/not met
Attaches syringe with saline and aspirates for blood and particles of marrow	met/not met
Slowly injects saline, observing for signs of infiltration	met/not met
Connects solution set and adjusts flow rate as necessary	met/not met
Disposes of sharps directly into the sharps container	met/not met
Attaches solution set and establishes patency of IV access	met/not met

**Table 2:** List of intraosseous access steps converted in the form of knowledge of performance and knowledge of results IO: intraosseous; KP: knowledge of performance; KR: knowledge of results

Step	KP version	KR version
1	The learner landmarks IO model 1-2 cm distal to tibial tuberosity on a flat portion of bone.	The learner landmarked.
2	The learner cleans the intended injection site in a circular motion out from the injection site using an alcohol wipe.	The learner disinfected the intended injection site.
3	The learner inserts the needle at approximately 90 degrees from the joint and stops when the bone is reached.	The learner inserted the needle.
4	The learner drills into the bone until "pop" is felt.	The learner drilled into the bone.
5	The learner secures the needle with a stabilizer so that there is no movement.	The learner stabilized the needle.
6	The learner attaches a 10 ml syringe filled with saline to the needle and aspirates for blood and particles of marrow.	The learner aspirated.
7	The learner slowly injects saline and monitors the drip.	Learner injected saline, so that a steady drip flowed from the end of the IO model, showing an indication of the correct injection site.

Delphi rounds

The seven steps identified in the DT were then subjected to consensus-building exercises following the Delphi methodology [16]. The results of this first Delphi round are shown in Table 3 below, where the frequencies, median, and standard deviation are noted.

**Table 3:** Results for Delphi Round 1 KP: knowledge of performance; KR: knowledge of results; SD: standard deviation

Step	KP / KR	Likert scale frequencies					Median	SD
		1	2	3	4	5		
1	KP	0	0	0	1	8	5	0.33
	KR	0	0	1	1	7	5	0.71
2	KP	0	0	1	2	6	5	0.73
	KR	0	0	2	0	7	5	0.88
3	KP	0	0	0	1	8	5	0.33
	KR	0	0	3	0	6	5	1
4	KP	0	0	0	2	7	5	0.44
	KR	0	0	3	0	6	5	1
5	KP	0	0	0	1	8	5	0.33
	KR	0	1	1	1	6	5	1
6	KP	0	1	2	0	6	5	1.2
	KR	1	1	3	1	3	3	1.42
7	KP	0	1	1	2	5	5	1.09
	KR	0	0	1	2	6	5	0.73

Based on the data from the first Delphi round, step six for both the KP and KR versions did not reach consensus (standard deviations were both above 1) and had to be fixed, as did the KP version of step seven since it did not meet the consensus criteria of having a standard deviation below 1. Comments from the participants are shown in Table 4. They indicated that aspirating for blood (step six) is no longer used in practice, so that step should be removed. They also noted that the KP for step seven was not worded properly and should be rewritten. Additionally, many participants noted that adding the step of preparing the patient as well as confirming the success of the procedure should be included as feedback as well.

**Table 4:** Comments from Delphi Round 1 KP: knowledge of performance; KR: knowledge of results; IO: intraosseous; IV: intravenous; FYI: for your information

Step	Comments
1	KR step one - I believe it should say "successfully landmarked" as it is possible to landmark incorrectly.
2	Circular motion is best practice but may not represent exactly what is occurring in the field.
3	Again, I believe 'where' the learner inserted the needle is a key component of the KR.
	Not a fan of the wording of the KP statement. Should read: "The learner inserts the needle through the skin at a 90-degree angle to the bone and stops when the bone is contacted."
	KR could be done incorrectly.
4	KR - Drilled into the bone in the correct area, ending in the osseous space without going through the bone.
	The KP and the KR statement are more than just drilling into the bone. The result should be the needle is drilled into the bone with consistent pressure and released when the pop is felt.
	KR may not have reached the correct location.
	The pop may not always be felt. Resistance is the key - resistance will lighten once in.
5	There really should be a step between four and five where the stylet is removed from the needle in the bone.
	Possibly the same result if the appropriate stabilizer device is used.
6	Aspirating for blood is no longer in our step-by-step process for confirmation, although it does confirm placement.
	Depending on where you read, this step may not be necessary. Recent procedures have steered away from this, as sometimes the bone marrow can block the IO needle.
	FYI, if a 10 ml syringe is filled with saline, there is no room to aspirate any material as the syringe is already full. The syringe could actually be empty for the purpose of aspiration.
	Doesn't always yield a positive result when done.
7	Although this is important, your initial flush is used to confirm patency and placement by determining if you 'don't' feel infiltration into the surrounding tissues. This might be hard in a simulated environment. You may be able to do this by creating a reservoir where saline can collect if the placement is not correct.
	The "flush" should be relatively quick to create the open space that allows the IV to drip afterwards.
	This is correct but can be worded that a saline flush is administered to ensure patency which is evidenced by the flow of liquid from the IO trainer.
	Not sure I understand this fully, but sounds as though this is to confirm the site, so it would be more important for the result than the actual skill of slowly infusing saline which is not part of IO insertion in practice
Overall	I think this is a minimal difference between the KP and the KR for a skill like this.
	Consider adding a final step speaking to confirm the IO procedure by ensuring the IV runs well, no signs of infiltration, no bruising or swelling of the leg etc.
	Maybe something related to prepping the patient (e.g., positioning).
	These all seem appropriate and would suggest technique and result are ideal for most of these steps for learning.
	You could have put something about indicating this procedure.

These results were then incorporated into the new steps listed in Table 5 below. In this new list of steps, step six was removed, two new steps were added, and the KP version of step seven was fixed, resulting in eight steps in the form of KP and KR. Only the new steps (steps one, seven, and eight in Table 3) went through another Delphi round with the same participants from round one and were given one week to complete. The results from the second Delphi round are shown in Table 6.

**Table 5:** Updated steps based on results of Delphi Round 1 KP: knowledge of performance; KR: knowledge of results

Step	KP version	KR version
1	The learner places the IO model on a sturdy surface and positions the IO model so it is externally rotated to display the medial aspect	The learner correctly placed and positioned the IO model
2	The learner landmarks IO model 1-2 cm distal to tibial tuberosity on a flat portion of bone	The learner successfully landmarked
3	The learner cleans the intended injection site in a circular motion out from the injection site using an alcohol wipe	The learner disinfected the intended injection site
4	The learner inserts the needle through the skin at a 90-degree angle to the bone, 1 cm to 2 cm inferior and medial to the tibial tuberosity in the flat portion of the tibia, and stops when the bone is contacted	The learner inserted the needle correctly, in the proximal tibia location
5	The learner drills into the bone in the proximal tibia location until resistance is lightened	The learner drilled into the bone in the proximal tibia location, ending in the osseous space, without going through the bone
6	The learner secures the needle with a stabilizer so that there is no movement	The learner stabilized the needle
7	The learner administers saline flush to ensure space for an IV drip	The learner administered saline flush, which is evidenced by the flow of liquid from the IO trainer
8	The learner attaches the solution set to the IO model and establishes the patency of IV access by monitoring the IV drip	The learner established the patency of IV access indicated by steady IV drip into the IO model

**Table 6:** Results from Delphi Round 2 KP: knowledge of performance; KR: knowledge of results; SD: standard deviation

Step	KP / KR	Likert scale frequencies					Median	SD
		1	2	3	4	5		
1	KP	0	2	0	2	4	5	1.11
	KR	0	2	1	3	2	4	1.07
7	KP	0	0	0	3	5	5	0.49
8	KP	0	0	1	2	4	5	0.79
	KP	0	0	1	2	4	5	0.79

The new step one did not reach consensus for both the KP and KR versions due to the standard deviation being above one. The comments are shown in Table 7 below, and they indicated that the step is important to indicate that the IO access simulator is positioned properly; however, indicating placement is not important as the simulator will already be on a steady surface; therefore, the step was reworded for KP and KR to satisfy the comments. The remainder of the steps met with consensus.

**Table 7:** Comments from Delphi Round 2 IO: intraosseous; KP: knowledge of performance; KR: knowledge of results

Step	Comments
1	I think the placement/display of the IO model on a sturdy surface may potentially impact the success of the remainder of the skill, however seeing as this "perfect" scenario never exists in the field, I don't think it's a valuable measure. Perhaps a more realistic scenario would be beneficial.
	It is important to comment on the external rotation as it will be difficult to perform the skill if the model isn't in the right position.
	The student must place the IO model on a hard surface so that he can train efficiently and safely.
7	I agree with your statement and understanding that saline access is used as placement confirmation. While other factors like feeling the "pop" or resistance met are confirmation methods directly related to the skill of IO insertion, I think this would be an important factor for an expert to evaluate the overall success of the skill performed. My other comparison would be the skill of intubation, and attaching equipment to see if lungs inflate - not directly related to the skill itself, but an important factor to evaluate. I hope that makes sense.
	I agree with the comments stating the flush is required to ensure patency and avoid infiltration into the surrounding tissues.
	The KP doesn't sound right to me, I believe it should state: The learner administers a saline flush and assesses for infiltration into the surrounding tissues. In real life, we can't see the fluid flow from the end of the model and therefore the KR would confirm you do not have infiltration (fluid in the surrounding tissues and not in the osseous space).
	It is important for the student to understand that this step (injecting saline and monitoring the drip) is important as it is part of successfully performing the procedure.
8	Typically the fluid would be placed under pressure.
	This is a good additional step - as it is a key component of initiating the IO.
	Step eight, we are actually looking at flow rate and it would be more important to look at the rate of flow because you may need to make a decision to use a pressure infuser to actually get a steady flow rate.
	It is important for the student to understand that this step (injecting saline and monitoring the drip) is important as it is part of successfully performing the procedure.

The resulting steps from the Delphi rounds are shown in Table 8. It is these steps that will be used by instructors to provide learners with KP and KR in the next study of this research to assess the perceived effectiveness of KP and KR on learning IO access using an IO access simulator. 

**Table 8:** Final list of steps with feedback in the forms of knowledge of performance and knowledge of results IO: intraosseous; KP: knowledge of performance; KR: knowledge of results

Step	KP version	KR version
1	The learner positions the IO model so it is externally rotated to display medial aspects	The learner correctly positioned the IO model
2	The learner landmarks IO model 1-2 cm distal to tibial tuberosity on the flat portion of bone	The learner successfully landmarked
3	The learner cleans the intended injection site in a circular motion out from the injection site using an alcohol wipe	The learner disinfected the intended injection site
4	The learner inserts the needle through the skin at a 90-degree angle to the bone, 1-2 cm inferior and medial to the tibial tuberosity in the flat portion of the tibia, and stops when the bone is contacted	The learner inserted the needle correctly, in the proximal tibia location
5	The learner drills into the bone in the proximal tibia location until resistance is lightened	The learner drilled into the bone in the proximal tibia location, ending in the osseous space, without going through the bone
6	The learner secures the needle with the stabilizer so that there is no movement	The learner stabilized the needle
7	The learner administers a saline flush and assesses for infiltration into the surrounding tissues	Learner administered saline flush
8	The learner attaches a solution set to the IO model, applies pressure, and establishes patency of IV access by monitoring the IV drip	The learner established the patency of IV access indicated by steady IV drip into the IO model

## Discussion

This discussion is organized around two main contributions: 1) pragmatic and 2) methodological. First, the purpose of this study was to determine what KP and KR can be provided to ACPs when learning IO access using an IO access simulator. To accomplish this, we followed the initial phase of a DBR approach, specifically within the design phase, using a hybrid of DT and Delphi methods. The main purpose of the design phase of DBR is to generate solutions that can address the theoretical and practical implications of a specific learning problem [8,10]. The theoretical concern we are addressing in this work is the matter of what type of feedback is perceived to be most effective by ACPs when learning IO access using an IO access simulator. When learning technical skills, such as IO access, feedback is the most important feature; however, it is still not known whether KP or KR is more effective [1,17]. Practically, we needed to determine what KP and KR can be given to learners, specifically in the context of learning IO access in an SBHPE environment. The DT session cultivated an initial list of steps, with each step being feedback that can be provided in the form of KP as well as KR. The Delphi rounds refined the list to result in eight steps, each written in the form of KP and KR so that it can be used as a guide for paramedic instructors to provide augmented feedback to ACPs learning IO access using an IO access simulator. In summary, the result was a set of steps when feedback needs to be provided. For each step, the experts and the learners provided input on how to operationalize the feedback to be either KR or KP in nature. This list will be used in subsequent research that will focus on testing and evaluating the perceived effectiveness of these types of feedback in training.

Second, the methodological contributions of this paper are centered around the use of the DBR approach in the construction of instructional (i.e., feedback) materials in SBHPE. Specifically, DBR was introduced to the educational field in the early 1990s by Ann Brown with the purpose of creating interventions in a collaborative manner (between the researcher and practitioner) that can be used in educational settings [13]. While this methodology has been predominantly used for traditional learning settings, with the increase in SBHPE, DBR can be applied to experiential learning as well. In an article by Schmitz et al. [18], a mobile simulation game was successfully designed and implemented following a DBR approach. Additionally, DBR has been used to create educational models for simulation facilitators [19]. There are no requirements for the methods used in each of the phases of DBR [9]. Therefore, using a combination of DT and Delphi methods to accomplish the design phase of DBR is unique to this research. However, it is not unique to SBHPE, as a study by Sivanathan et al. [20] has used a combination of DT and Delphi to help guide the design of a virtual reality simulation to help with moral distress experienced by healthcare professionals. As DBR necessitates the cooperation of researchers, designers, educators, and learners, employing a blend of DT and Delphi methods offers a chance to fulfill the requirements of all involved parties. For example, designers, educators, and learners prefer utilizing DT, a creative design process that facilitates idea generation and problem-solving. Conversely, the use of the Delphi method satisfies the rigorous demands of researchers seeking approaches that ensure content validity. Therefore, using a combination of the two approaches allows for the creative generation of solutions that can be validated through consensus-building [21].

There are a few limitations and strengths to this study. First, while DT and Delphi methods are validated methodological approaches, using them in combination with each being modified has not been validated as an approach. Despite this combination being used successfully in the past [20,21] for SBHPE scenarios, the approach itself has not been tested and validated. On the other hand, this methodological combination is unique to DBR and can be an advancement for the field. One of the strengths of this work is that we were able to get the perspectives and feedback of stakeholders (paramedic instructors) as well as end-point users (ACPs and ACP students). However, our small sample size for the Delphi rounds was a limitation, as a sample of 15-30 is more adequate [16].

## Conclusions

In this article, the utilization of the DBR framework integrating the DT and Delphi methods is described. The primary objective of this study was to determine what KP and KR can be provided by paramedic instructors to ACPs in the context of learning IO access skills using a previously developed IO access simulator. Through the systematic application of the DT method, an initial list of feedback in the form of KP and KR was developed based on an IO access skills checklist. The Delphi method provided a consensus on the identified KP and KR for instructing ACPs in IO access skills. This resulted in an eight-step list of feedback in the form of KP and KR, which will be used in the next phase of this research project, which assesses the perceived effectiveness of feedback in training ACPs IO access skills in an SBHPE environment.

## References

[1] Schmidt RA, Lee TD, Winstein CJ (2018). Motor control and learning: a behavioral emphasis (6th ed.). https://books.google.co.in/books?hl=en&lr=&id=EvJ6DwAAQBAJ&oi=fnd&pg=PR1&dq=Motor+control+and+learning:+a+behavioral+emphasis+(6th+ed.)&ots=k5IsbHs9NI&sig=Wkb5B2MKdIW2G82isKvr8BfP99k&redir_esc=y#v=onepage&q=Motor%20control%20and%20learning%3A%20a%20behavioral%20emphasis%20(6th%20ed.)&f=false.

[2] Dubrowski A, Backstein D (2004). The contributions of kinesiology to surgical education. J Bone Joint Surg Am.

[3] Chiniara G, Cole G, Brisbin K, Huffman D, Cragg B, Lamacchia M, Norman D (2013). Simulation in healthcare: a taxonomy and a conceptual framework for instructional design and media selection. Med Teach.

[4] Dubrowski A, Kapralos B, Peisachovich E, Da Silva C, Torres A (2021). A model for an online learning management system for simulation-based acquisition of psychomotor skills in health professions education. Cureus.

[5] Kothari LG, Shah K, Barach P (2017). Simulation based medical education in graduate medical education training and assessment programs. Prog Pediatr Cardiol.

[6] Sivanathan M, Micallef J, Clarke KM (2022). The development and initial end-point user feedback of a 3D-printed adult proximal tibia IO simulator. Cureus.

[7] Fahd K, Miah SJ, Ahmed K (2021). Integrating design science research and design based research frameworks for developing education support systems. Educ Inf Technol (Dordr).

[8] Wang F. Hannafin MJ (2005). Design-based research and technology-enhanced learning environments. Educ Technol Res Dev.

[9] Scott EE, Wenderoth MP, Doherty JH (2020). Design-based research: a methodology to extend and enrich biology education research. CBE Life Sci Educ.

[10] Edelson DC (2002). Design research: what we learn when we engage in design. J Learn Sci.

[11] Hoadley CM (2004). Methodological alignment in design-based research. Educ Psychol.

[12] Barab S, Squire K (2004). Design-based research: putting a stake in the ground. J Learn Sci.

[13] Anderson T, Shattuck J (2012). Design-based research: a decade of progress in education research?. Educ Res.

[14] Cobb P, Confrey J, DiSessa A (2003). Design experiments in educational research. Educ Res.

[15] McLaughlin JE, Wolcott MD, Hubbard D, Umstead K, Rider TR (2019). A qualitative review of the design thinking framework in health professions education. BMC Med Educ.

[16] Haji FA, Khan R, Regehr G, Ng G, de Ribaupierre S, Dubrowski A (2015). Operationalising elaboration theory for simulation instruction design: a Delphi study. Med Educ.

[17] Sharma DA, Chevidikunnan MF, Khan FR, Gaowgzeh RA (2016). Effectiveness of knowledge of result and knowledge of performance in the learning of a skilled motor activity by healthy young adults. J Phys Ther Sci.

[18] Schmitz B, Klemke R, Walhout J (2015). Attuning a mobile simulation game for school children using a design-based research approach. Comput Educ.

[19] Koivisto JM, Hannula L, Bøje RB, Prescott S, Bland A, Rekola L, Haho P (2018). Design-based research in designing the model for educating simulation facilitators. Nurse Educ Pract.

[20] Sivanathan M, Espinola CW, Uribe Quevedo A, Kapralos B, Krishnan S, Bhat V, Dubrowski A (2022). Development of content for a virtual reality simulation to understand and mitigate moral distress in healthcare workers. Cureus.

[21] Sivanathan M, Yanguez Franco L, Joshi S, Micallef J, Button D, Dubrowski A (2022). Development of simple and advanced adult proximal tibia simulators for a decentralized simulation-based education model to teach paramedics-in-training the intraosseous infusion procedure. Cureus.

